# Alternative splicing at NAGNAG acceptors in *Arabidopsis thaliana *SR and SR-related protein-coding genes

**DOI:** 10.1186/1471-2164-9-159

**Published:** 2008-04-10

**Authors:** Stefanie Schindler, Karol Szafranski, Michael Hiller, Gul Shad Ali, Saiprasad G Palusa, Rolf Backofen, Matthias Platzer, Anireddy SN Reddy

**Affiliations:** 1 Genome Analysis, Leibniz Institute for Age Research – Fritz Lipmann Institute, Beutenbergstr. 11, 07745 Jena, Germany; 2 Institute of Computer Science, Bioinformatics Group, Albert-Ludwigs-University Freiburg, Georges-Koehler-Allee 106, 79110 Freiburg, Germany; 3 Department of Biology and Program in Molecular Plant Biology, Colorado State University, Fort Collins, CO, USA

## Abstract

**Background:**

Several recent studies indicate that alternative splicing in Arabidopsis and other plants is a common mechanism for post-transcriptional modulation of gene expression. However, few analyses have been done so far to elucidate the functional relevance of alternative splicing in higher plants. Representing a frequent and universal subtle alternative splicing event among eukaryotes, alternative splicing at NAGNAG acceptors contributes to transcriptome diversity and therefore, proteome plasticity. Alternatively spliced NAGNAG acceptors are overrepresented in genes coding for proteins with RNA-recognition motifs (RRMs). As SR proteins, a family of RRM-containing important splicing factors, are known to be extensively alternatively spliced in Arabidopsis, we analyzed alternative splicing at NAGNAG acceptors in SR and SR-related genes.

**Results:**

In a comprehensive analysis of the *Arabidopsis thaliana *genome, we identified 6,772 introns that exhibit a NAGNAG acceptor motif. Alternative splicing at these acceptors was assessed using available EST data, complemented by a sequence-based prediction method. Of the 36 identified introns within 30 SR and SR-related protein-coding genes that have a NAGNAG acceptor, we selected 15 candidates for an experimental analysis of alternative splicing under several conditions. We provide experimental evidence for 8 of these candidates being alternatively spliced. Quantifying the ratio of NAGNAG-derived splice variants under several conditions, we found organ-specific splicing ratios in adult plants and changes in seedlings of different ages. Splicing ratio changes were observed in response to heat shock and most strikingly, cold shock. Interestingly, the patterns of differential splicing ratios are similar for all analyzed genes.

**Conclusion:**

NAGNAG acceptors frequently occur in the Arabidopsis genome and are particularly prevalent in SR and SR-related protein-coding genes. A lack of extensive EST coverage can be compensated by using the proposed sequence-based method to predict alternative splicing at these acceptors. Our findings indicate that the differential effects on NAGNAG alternative splicing in SR and SR-related genes are organ- and condition-specific rather than gene-specific.

## Background

Alternative splicing is an important mechanism for regulating gene expression at the post-transcriptional level and contributes to proteome complexity [1-3]. This widespread process comprises various mechanisms such as exon skipping, mutually exclusive exons, intron retention, or the usage of alternative 5' or 3' splice sites [4]. Alternative splicing has been extensively studied in mammals but less in plants. Recent evidence indicates more than 60% of the genes in the human genome alternatively spliced [5] compared to about 20–30% in plants [6,7], based on EST/cDNA data. A hallmark of plant introns is their relatively short length (~150 vs. ~740 nt in humans, on average) [8] and Uridine-richness [9]. Furthermore, plant introns exhibit a weaker polypyrimidine tract than mammals [2,9]. Datasets of spliced alignments from the TIGR [6,10] and RIKEN [11] databases of full-length cDNAs and ESTs provide useful annotated versions of the Arabidopsis genome sequence for the detection of various alternative splice events. Based on the final TIGR annotation release, a total of 26,207 genes are annotated in Arabidopsis [12]. Although splicing machinery is generally conserved between plants and animals [2,9], plants exhibit a much higher fraction of retained introns (more than 40% of the events) compared to ~10% reported for humans [5-8].

The fidelity of intron excision from a pre-mRNA relies on the precise recognition of exonic and intronic sequence signals and the complex interplay of different spliceosomal RNAs and proteins. Among these, SR proteins direct splice site selection by recognizing splice sites and splicing regulatory sequences (enhancers and silencers), thereby facilitating spliceosome assembly [13,14]. SR proteins are important factors for constitutive and alternative splicing. This evolutionary conserved protein family contains structurally related proteins that possess one or two RNA-recognition motifs (RRM) at the N-terminus and a C-terminal arginine/serine-rich (RS) domain [15,16]. A recent genome-wide survey on Arabidopsis splicing-related genes revealed variations in SR proteins and hnRNP proteins between plants and mammals, suggesting plant-specific differences in splicing-regulation mechanisms [17]. The *A. thaliana *genome encodes 19 SR proteins, almost twice as many as in humans [18,19]. They can be subdivided into seven families [20]. Whereas SF2/ASF, 9G8 and SC35 are orthologues between plants and metazoa, the RS, RS2Z, SCL and SR45 subfamilies seem to be plant-specific. Most of the SR protein genes are themselves alternatively spliced to a great extent in Arabidopsis [20,21]. Fifteen of the 19 genes coding for SR proteins in Arabidopsis undergo alternative splicing and produce at least 95 transcripts [21]. In some cases, it was shown that alternative splicing correlates with the intron length [22]. Splicing patterns of Arabidopsis SR protein genes are under tight spatio-temporal control, leading to a different abundance of splice variants in different tissues and at developmental stages [21,23-26]. Several plant SR proteins have been shown to regulate the splicing of their own transcripts and transcripts of other SR genes [25,27-29]. Environmental conditions can also modulate the splicing pattern of a gene, as shown by the temperature dependent alternative acceptor selection of *SR1B/SR1 *in Arabidopsis [30]. Furthermore, stresses such as exposure to cold, heavy metals or anaerobiosis, affect the efficiency or patterns of splicing [21,31-33], but the mechanisms by which some types of stress influence alternative splicing in plants are largely unknown.

In plants and mammals, the most frequent distance between alternative acceptors is 3 nt [6,34]. Such tandem acceptors have been termed NAGNAG acceptors based on the existence of a NAGNAG acceptor motif (N = A, C, G, T) [35,36]. In the NAGNAG motif, the upstream acceptor is termed the E-acceptor (since the downstream NAG becomes exonic upon splicing at this site) and the downstream one the I-acceptor (since the whole tandem becomes intronic) [36]. Alternative splicing at NAGNAG acceptors is widespread in many species [37,38] and also in plants [6,39] with *Caenorhabditis elegans *[36] being the only exception known so far. The selection of either AG in the splicing process results in the insertion/deletion (indel) of the I-acceptor NAG in mRNAs. This leads to diverse effects at the protein level with the majority of the events involving the indel of a single amino acid. A fraction of these events is estimated to be under purifying selection, suggesting an evolutionary conserved function [40]. Interestingly, it was demonstrated that the distribution of NAGNAG acceptors is highly similar between mammals and plants, for example, polar amino acid residues were found to be predominantly affected in both kingdoms [36,41].

An example for functional alternative splicing in Arabidopsis is a TAGCAG acceptor affecting the RNA-binding domain of the U11–35K protein that results in different binding affinity for SR proteins and the U11 snRNA *in vitro *[42]. In contrast, both splice variants derived from a CAGCAG acceptor in the tomato prosystemin gene are active signaling components of the wound response pathway, without detectable functional differences [39].

Previously, we found that human genes coding for RNA binding proteins including many splicing factors are preferentially equipped with NAGNAG acceptors [36]. Here, we observed a similar overrepresentation of NAGNAG acceptor motifs in Arabidopsis. This agrees with a very recent study, where NAGNAG alternative splicing was also found to be accumulated in genes for RNA-binding proteins in Arabidopsis [41].

Since splice variants of splicing factors may have consequences for alternative splicing and its regulation [43], we investigated alternative NAGNAG splicing at SR and SR-related genes. We determined the splicing ratios of 15 NAGNAG acceptors in splicing factors for several plant tissues, seedlings of different ages and in response to cold and heat stresses. We detected organ-specific variations and differences between the developmental stages. Cold stress was found to induce the most remarkable changes in the splicing ratios.

## Results

### NAGNAG acceptors are frequent in the Arabidopsis genome

A comprehensive list of introns was constructed from the annotated Arabidopsis genome sequence based on RIKEN [11] and TIGR [6,10] cDNA sequences. Out of 112,934 intron-exon boundaries (taken from 26,207 annotated protein-coding genes), 6,772 showed a NAGNAG motif within 5,381 genes (Additional table 1). Thus, 6% of all introns and 21% of all annotated genes in Arabidopsis harbor a genomic NAGNAG acceptor motif. For comparison, in human, 5% of introns and 30% of genes harbor such a motif [36]. We categorized all Arabidopsis cases according to their EST coverage (Additional table 2). In 229 cases (3%), no EST support exists for either of the possible acceptor sites. In 1,899 cases (28%), a single EST supports either acceptor. Out of the remaining 4,644 cases with minimally required EST coverage (two or higher, 69%), 242 cases (5%) have supporting ESTs for both acceptor sites. Naturally, EST-based evidence for alternatively spliced NAGNAGs depends on their isoform frequencies and the EST coverage, which is low in Arabidopsis compared to other species such as human or mouse. For example, if a minor isoform occurs with 10% frequency, at least 29 ESTs are necessary to reach a probability of 95% that it will be found (binomial test). Hence, in many cases, native alternative splicing remains undetected, and certainly more NAGNAG sites than those indicated by the current transcript data are expected to be alternatively spliced.

In order to overcome this limitation of EST coverage we established a sequence-based prediction method for alternative splicing at NAGNAG acceptors. There is evidence that a narrow context of flanking nucleotides captures most of the information relevant for prediction of the splice variant ratio [44,45]. Conservatively, we chose a heptameric context NAGNAGN, comprising the two acceptor AG dinucleotides and three additional variable positions, and divided all NAGNAG cases into 64 heptamer classes. The EST counts within each of the classes were pooled, and the resulting splicing variant ratio (fraction of E-transcripts) was considered representative for all cases of that heptamer class. For example, the average frequency of the E-isoforms of 55 observed CAGCAGA acceptors, based on 227 pooled ESTs, is 48%, and this was taken as the predicted frequency for any CAGCAGA acceptor motif (Additional Table 3). The validity of the heptamer-based approach is corroborated by the finding that maximum-likelihood estimators mostly agree between models for Arabidopsis and human. The high level of agreement is explained by the basic finding that the splicing ratios follow the basic rules of sequence preferences seen for isolated 3' splice sites: position -3 with C ≥ T > A > G, position +1 with G ≥ A > T > C (data not shown).

Applying this method to the 2,128 cases with insufficient EST coverage (less than two ESTs), 482 (23%) are predicted to have a minor transcript frequency of at least 10%. Using this conservative threshold for isoform abundance gives a lower-bound estimate of the fraction of alternatively spliced NAGNAG sites. Applying this prediction method to all NAGNAG cases in Arabidopsis, 14% are predicted to be alternatively spliced with a minor transcript frequency of at least 10%, 21% with ≥ 5%, and 33% with ≥ 2%, respectively. Interestingly, as EST coverage increases, NAGNAG acceptors are less often predicted to be alternatively spliced (<2 ESTs: 23%, 2–5 ESTs: 11%, >5 ESTs: 8%). These results indicate that the occurrence of alternatively spliced NAGNAG acceptors is negatively correlated with the transcript levels of the genes.

### Many SR and SR-related protein transcripts contain NAGNAG acceptors

For identification of SR and SR-related genes we searched for characteristic protein signatures in the gene products associated with NAGNAG acceptors [46]. Of all Arabidopsis proteins, 84 proteins had RRM domains and are rich for R/S dipeptides. Of these 84, 19 were previously identified as SR proteins [18,19], leaving 65 SR-related proteins. The intersection with NAGNAG cases gave 36 introns in 30 genes (Table 1). Thus, 36% of SR and SR-related protein-coding genes exhibit NAGNAG acceptors (7 out of 19 SR, 23 out of 65 SR-related). This is significantly higher than the average frequency of NAGNAG-containing genes (21%), even if we account for a higher fraction of multi-exon genes and a slightly higher number of introns in the SR/SR-related gene family (P = 0.068, permutation test). This finding is very similar to human where alternatively spliced NAGNAG motifs were found to be enriched in RRM-containing proteins [36].

**Table 1:** NAGNAG acceptors in Arabidopsis SR and SR-related protein-coding genes. In summary, 36 NAGNAG-containing introns occur in 30 genes. Genes are classified into SR and SR-related protein coding genes. Splicing ratios are given as absolute EST counts ("#"). Column 'heptamer motif' specifies the heptamer sequence of the NAGNAG acceptor sites used for the sequence-based prediction; here, "|" marks the annotated acceptor. Predicted E-transcript proportions are listed in column 'E-transcript predicted'. Gene names are grey shaded if they contain two NAGNAG acceptors.

**Gene**	**Name**	**SR**	**SR-related**	**Intron**	**# ESTs E-transcript**	**# ESTs I-transcript**	**Heptamer motif**	**E-transcript predicted (%)**
At5g52040	*RS41*	*x*		2	5	17	AAG|CAG,G	7
At4g31580	*RSZ22*	*x*		3	15	0	TAG|GAG,C	99
At3g13570	*SCL30a*	*x*		3	13	0	TAG|GAG,G	99
At1g55310	*SR33/SCL33*	*x*		3	0	8	CAG,CAG|A	48
At1g16610	*SR45*	*x*		7	0	12	CAG,CAG|G	48
At1g16610	*SR45*	*x*		9	0	12	AAG,CAG|G	7
At1g23860	*SRZ21*	*x*		3	13	0	CAG|GAG,A	100
At2g24590	*SRZ22a*	*x*		3	8	0	CAG|AAG,A	98
At1g07350			*x*	2	21	0	TAG|GAG,A	100
At1g22910			*x*	1	7	0	TAG|GAG,C	99
At1g53650	*CID8*		*x*	4	7	0	CAG|AAG,G	97
At1g60000	*cp29*		*x*	1	6	0	CAG|GAG,T	100
At1g60900	*U2AF65*		*x*	2	3	0	CAG|GAG,A	100
At1g60900	*U2AF65*		*x*	4	0	2	TAG,CAG|G	17
At1g76940			*x*	1	2	0	TAG|AAG,G	83
At2g24350			*x*	4	0	2	AAG,CAG|T	7
At2g24350			*x*	1	5	0	CAG|GAG,T	100
At2g35410	*cp33*		*x*	3	16	0	TAG|GAG,T	100
At2g37220	*cp29*		*x*	2	22	0	TAG|GAG,T	100
At2g43370	*U11–35K*		*x*	4	0	6	TAG,CAG|G	17
At2g43370	*U11–35K*		*x*	5	5	0	CAG|GAG,T	100
At3g23830	*GR-RBP4/GRP4*		*x*	3	15	0	CAG|GAG,T	100
At3g26420	*ATRZ-1A*		*x*	3	5	0	TAG|AAG,G	83
At3g51950			*x*	4	6	0	CAG|GAG,G	100
At3g54230			x	5	0	3	GAG,CAG|G	1
At3g54230			*x*	6	0	3	TAG,CAG|C	25
At4g35785			*x*	4	0	0	CAG,AAG|T	98
At4g36960			*x*	8	5	0	TAG|GAG,A	100
At5g02530			*x*	2	6	0	TAG|GAG,A	100
At5g03580	*PABP*		*x*	1	0	1	GAG,TAG|G	0
At5g09880			*x*	1	5	0	CAG|AAG,A	98
At5g44200	*CBP20*		*x*	1	4	0	CAG|GAG,A	100
At5g44200	*CBP20*		*x*	7	6	0	CAG|GAG,G	100
At5g47320	*RPS19*		*x*	3	8	0	TAG|GAG,T	100
At5g53180	*PTB*		*x*	7	0	7	GAG,TAG|G	0
At5g59950			*x*	1	0	11	TAG,CAG|A	7

*SR33/SCL33 *is the only case which exhibits EST support for alternative NAGNAG splicing (Table 1). Intriguingly, in 14 cases, the sequence-based prediction argues for the usage of both acceptor sites with a predicted minor transcript frequency of 2%. This permissive 2% threshold was applied in narrowing the list of experimental candidates in order to retain those which have a substantial chance to be alternatively spliced. We selected 15 SR and SR-related protein-coding genes for experimental analysis, including *SR33/SCL *as a positive control (Table 2).

**Table 2:** Experimental candidates with corresponding E-transcript proportions based on EST and experimental data. EST ratios are given as absolute EST counts. Column 'predicted' lists the E-transcript proportions obtained from the sequence-based prediction. Grey shaded values mark the cases where NAGNAG alternative splicing was validated (avg [minor isoform in organs, seedlings] > 2× avg [error]). At4g35785 is lacking EST data. Therefore, an EST-based E-transcript frequency cannot be shown and is indicated by '-'.

	**E-transcript (%)**		
**Gene**	**EST**	**Predicted**	**Experiments**

*RS41*	23	7	16.7 ± 0.6
*SR33/SCL33*	0	48	29.9 ± 0.7
*SR45i7*	0	48	0.6 ± 0.5
*SR45i9*	0	7	0.4 ± 0.6
*SRZ22a*	100	98	99.5 ± 0.6
*CID8*	100	97	99.1 ± 0.5
At1g76940	100	81	98.8 ± 0.9
At2g24350	0	7	3.2 ± 1.5
*ATRZ-1A*	100	83	98.9 ± 1.4
At3g54230	0	25	2.3 ± 0.5
At4g35785	-*	98	97.4 ± 0.3
At5g09880	100	98	99.0 ± 0.7
At5g59950	0	7	2.8 ± 0.4
*U11–35K*	0	17	8.5 ± 0.9
*U2AF65*	0	17	5.8 ± 0.6

### Experimental evidence for NAGNAG isoforms in SR and SR-related protein genes

For experimental detection of splice variants, cDNA from different adult plant organs (root, leaf, stem, inflorescence) and from callus and seedlings of different ages (3d, 5d, 10d, 15d) was sampled to cover a broad spectrum of transcript sources. Three independent RT-PCRs were performed per cDNA sample and gene, and splice variants were separated by capillary electrophoresis and subsequently quantified based on fluorescence intensity. We considered a NAGNAG candidate as alternatively spliced if the measurements indicated an average minor transcript frequency of at least two times the standard deviation. In a conservative approach, we evaluated the averages of the plant samples, in order to avoid extreme values from single samples that could cause false positives.

Eight cases of SR and SR-related protein-coding genes were found to be alternatively spliced at their NAGNAG acceptor sites (53% of 15 tested, 22% of NAGNAGs in this family; Table 2, Additional Table 4). In addition, the alternative splicing patterns of *RS41 *and *SR33 *were independently confirmed by Sanger sequencing of at least 100 clones (data not shown). Using the same approach, alternative NAGNAG splicing could not be detected in *SR45i9 *and *SRZ22a*, consistent with the quantitative capillary electrophoresis results. It is noteworthy that the relatively high frequency of alternative splicing in *SR33/SCL33 *was not indicated by the eight ESTs that exist for that transcript region.

In the tested cases, the E-acceptor was found to represent the minor acceptor in nearly all cases, and the prediction for alternative splicing was more often accurate for the major-I subclass compared to major-E subclass. This is consistent with the global case distribution evident from EST data, which divides into 13% constitutive I, 17% alternative major-I, 13% alternative major-E, 57% constitutive E cases (based on the 2%-abundance threshold).

Genome-wide, the sequence-based prediction method suggested that 33% of NAGNAGs are alternatively spliced, producing minor isoforms with at least 2% frequency. For the 15 SR/SR-related cases that fulfill this prediction criterion, 53% were actually validated by our experiments. The prediction accuracy is positively correlated with the predicted minor transcript frequency. For example, cases predicted to have 2–5% minor transcripts are validated with a rate of 25% whereas cases predicted to have more than 5% minor transcripts are validated with a rate of 64% (Table 2). Consequently, a threshold of 2% seems to fully capture the fraction of likely alternatively spliced NAGNAG acceptors, as was intended for the selection of experimental candidates. Unfortunately, independent measures for the fraction of non-SR protein genes that undergo alternative NAGNAG splicing do not exist. However, based on the prediction results, we expect that SR/SR-related protein genes have a slightly higher propensity for alternative splicing (42% versus 33%).

### Organ-specific alternative splicing of NAGNAG acceptors and differential splicing ratios during development

**Figure 1:**
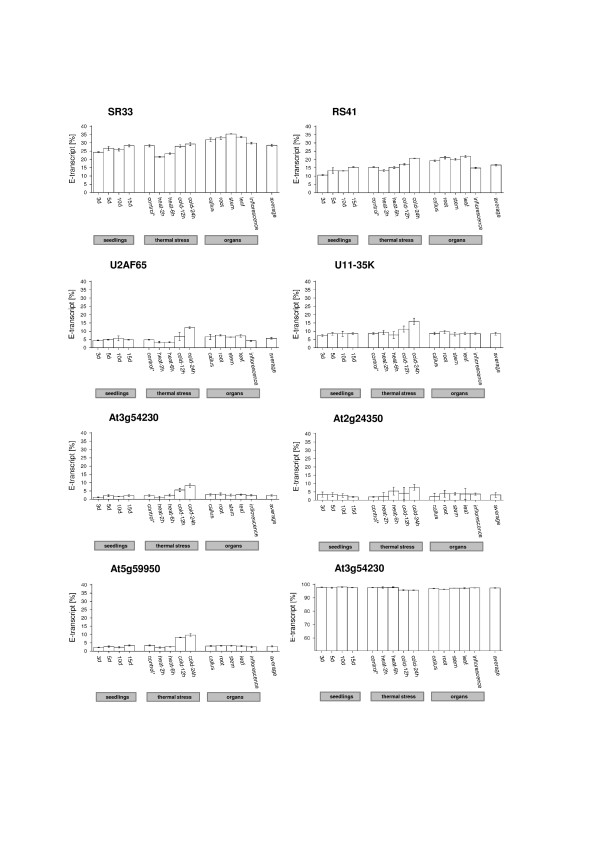
**E-transcript proportions in SR and SR-related protein-coding genes under several conditions**. E-transcript proportions among different seedling ages, under heat and cold shock and in different organs are depicted. The 15d old seedlings also serve as the control for the thermal stress treatments, indicated by '*'.

Splicing patterns of Arabidopsis SR protein genes are under tight spatio-temporal control, leading to a different abundance of splice variants in different tissues and at developmental stages [21,23-26]. Thus, we considered the occurrence of possible differences in the splice variant distribution in various plant organs (root, leaf, stem and inflorescence) and in callus. Based on the prior results, we similarly tested the cDNAs from those candidates, where the NAGNAG alternative splicing was successfully validated. In four cases (Figure 1, Table 3), a significant organ-specificity was observed (ANOVA, Table 3). Interestingly, inflorescence tissue shows reduced splicing of the minor acceptor (mostly E-acceptor) in nearly all cases. This trend is also observed for the NAGNAG cases that do not show significant organ-specific splicing.

**Table 3:** E-transcript proportions in plant organs. The E-transcript frequencies in plant organs (root, leaf, stem, inflorescence) and callus are illustrated. Values were obtained from three independent experiments. Column 'ANOVA' displays the p-values, '+' p ≤ 0.05 and '++' p ≤ 0.01.

	**E-transcript(%)**					
**Gene**	**Callus**	**Root**	**Stem**	**Leaf**	**Inflor**	**ANOVA**

*RS41*	19.4 ± 0.4	21.1 ± 0.8	20.2 ± 0.6	22.0 ± 0.7	14.9 ± 0.5	++
*SR33/SCL33*	31.8 ± 1.2	33.1 ± 0.9	35.4 ± 0.2	33.5 ± 0.3	29.8 ± 0.6	++
At2g24350	2.0 ± 2.2	3.8 ± 2.1	3.9 ± 0.8	3.7 ± 3.5	3.6 ± 0.7	
At3g54230	2.7 ± 0.7	3.0 ± 0.8	2.5 ± 0.7	2.8 ± 0.4	2.4 ± 0.4	
At4g35785	97.0 ± 0.2	96.3 ± 0.2	97.2 ± 0.1	97.2 ± 0.5	97.5 ± 0.2	+
At5g59950	3.0 ± 0.3	3.2 ± 0.3	3.2 ± 0.4	3.0 ± 0.5	2.5 ± 0.5	
*U11–35K*	8.7 ± 0.9	9.6 ± 1.0	8.3 ± 1.2	8.8 ± 0.7	8.6 ± 0.5	
*U2AF65*	6.5 ± 1.6	7.6 ± 0.5	6.5 ± 0.1	7.2 ± 0.8	4.2 ± 0.3	+

Next, we asked if the splicing ratios at the NAGNAG acceptors exhibit developmental variations. To this end, we analyzed cDNA derived from seedlings at the ages of 3d, 5d, 10d and 15d (Table 4, Figure 1). In four analyzed cases, statistically significant splicing ratio changes could be detected (ANOVA, Table 4). Comparing the values of the 3d, 5d, 10d and 15d probes, our results show a general trend towards increased minor acceptor usage with seedling development.

**Table 4:** E-transcript proportions among different developmental stages. The E-transcript frequencies in seedlings of different ages (3d, 5d, 10d, 15d) are presented. Values were obtained from three independent experiments. Column 'ANOVA' displays the p-values, '+' p ≤ 0.05 and '++' p ≤ 0.01.

	**E-transcript(%)**				
***Gene***	**3d**	**5d**	**10d**	**15d**	**ANOVA**

*RS41*	10.6 ± 0.4	13.3 ± 1.8	13.1 ± 0.2	15.4 ± 0.3	+
*SR33/SCL33*	24.4 ± 0.3	26.7 ± 1.3	25.9 ± 0.8	28.2 ± 0.7	+
At2g24350	3.3 ± 1.6	3.3 ± 1.3	2.8 ± 1.1	1.9 ± 0.4	
At3g54230	1.2 ± 0.3	2.2 ± 0.6	1.8 ± 0.2	2.3 ± 0.6	+
At4g35785	97.9 ± 0.2	97.6 ± 0.5	98.1 ± 0.2	97.7 ± 0.3	
At5g59950	2.2 ± 0.3	2.6 ± 0.5	2.4 ± 0.5	3.4 ± 0.4	+
*U11–35K*	7.3 ± 0.8	8.5 ± 0.8	8.4 ± 1.6	8.6 ± 0.6	
*U2AF65*	4.4 ± 0.3	4.9 ± 0.3	5.7 ± 1.6	4.8 ± 0.2	

### NAGNAG splicing ratios under heat and cold shock

Finally, we examined whether temperature stresses can modulate the NAGNAG splicing pattern, as was previously illustrated by temperature-controlled splicing ratios of *SRp34/SR1 *and other SR transcripts [21,30]. Seedlings were kept in hot or cold conditions and compared to an untreated control. Rather slight splicing ratio changes could be observed in the heat-shocked probes (Figure 1, Table 5). However, this difference was statistically significant in four cases (ANOVA, Table 5). In contrast, more obvious splicing ratio changes could be detected under cold shock (Figure 1). In six cases, a significant rise in minor acceptor usage was observed (ANOVA, Table 5), that clearly increased with the duration of treatment.

**Table 5:** E-transcript proportions under heat and cold shock. The E-transcript frequencies of seedlings kept in hot and cold conditions (2 h vs. 6 h and 12 h vs. 24 h, respectively) compared to untreated seedlings. Values were obtained from three independent experiments. Column 'ANOVA' displays the p-values, '+' p ≤ 0.05 and '++' p ≤ 0.01.

	**E-transcript(%)**					**ANOVA**	
***Gene***	**Untreated control**	**Heat shock 2 h**	**Heat shock 6 h**	**Cold shock 12 h**	**Cold shock 24 h**	**Heat shock**	**Cold shock**

*RS41*	15.4 ± 0.3	13.2 ± 0.6	15.3 ± 0.8	17.3 ± 0.6	20.7 ± 0.2	+	++
*SR33/SCL33*	28.2 ± 0.7	21.5 ± 0.4	23.5 ± 0.5	28.0 ± 0.9	29.3 ± 1.0	++	
At2g24350	1.9 ± 0.4	2.0 ± 2.4	5.4 ± 2.5	4.1 ± 3.6	7.7 ± 1.7		
At3g54230	2.3 ± 0.6	1.0 ± 0.9	2.5 ± 0.7	5.6 ± 1.1	8.2 ± 1.1		+
At4g35785	97.7 ± 0.3	97.8 ± 0.6	97.8 ± 0.4	95.8 ± 0.6	95.7 ± 0.2		+
At5g59950	3.4 ± 0.4	2.1 ± 0.5	2.5 ± 0.2	8.2 ± 0.3	9.6 ± 0.8	+	++
*U11–35K*	8.6 ± 0.6	9.4 ± 1.2	7.8 ± 2.0	11.3 ± 1.8	16.0 ± 2.0		+
*U2AF65*	4.8 ± 0.2	3.5 ± 0.6	3.4 ± 0.4	6.9 ± 2.6	12.1 ± 0.6	+	+

## Discussion

SR proteins are important regulatory splicing factors and facilitate the correct interplay of components of the splicing machinery. Alternative splicing of SR protein genes is able to confer a spatial flexibility to the architecture of the spliceosome and thus may influence the splicing process and its outcome. Subtle changes in the protein composition induced by alternative splicing at NAGNAG acceptors could contribute to this flexibility as previously suggested [36]. Here we explored the degree of alternative splicing at NAGNAG acceptors in Arabidopsis in general and of SR and SR-related protein-coding genes in detail. In a genome-wide *in silico *screening we identified 6,772 introns that exhibit a NAGNAG acceptor motif. Out of this group, we identified 36 introns within 30 SR and SR-related protein-coding genes. Intriguingly, NAGNAG acceptor motifs are more frequent in Arabidopsis SR and SR-related protein-coding genes (36%) than on average (21%). This is equivalent to the situation in human [36].

EST and mRNA data are the main resources to identify and locate alternative splicing of a gene. The total number of ESTs for a respective gene correlates with the diagnostic power of ESTs. Due to the relatively low EST coverage of the Arabidopsis genome, the EST data alone is not sufficient for a comprehensive characterization of the alternative splicing of NAGNAG motifs. For example, guided by the sequence-based prediction we could experimentally show an E-transcript frequency of 30% for *SR33/SCL33 *despite initially lacking EST evidence for alternative splicing. This illustrates that the limitations of a low EST coverage can be at least in part circumvented with an appropriate prediction method. Currently, the EST data provides evidence for alternative splicing of 5% (242 cases) of NAGNAG acceptors, which represents the lower bound for genome-wide estimates. On the other hand, our sequence-based prediction method suggested 33% of genes produce minor isoforms with at least 2% frequency. But these predictions were found to be too optimistic, with only 53% of the cases actually giving detectable amounts of NAGNAG isoforms. Extrapolating these results to genome scale, 17% of Arabidopsis NAGNAGs are likely to be alternatively spliced. All prediction work neglects possible differences between tissues or developmental stages. In fact, our results for SR and SR-related protein genes indicate that organ- and development-specific, as well as stress-induced differences exist. However, the mechanisms that underlie tissue-specific regulation of alternative splicing are not yet understood and are not predictable by any current method.

We found a negative correlation of the occurrence of alternatively spliced NAGNAG acceptors with the transcript levels of the genes. Though this finding needs further validation, it suggests that genes with high transcript abundance are not representative for the transcriptome. This would have profound consequences for studies extrapolating from highly expressed genes to the remaining transcriptome.

The effects of splicing factors are often dependent on their concentration, localization and phosphorylation, resulting in gradual changes of the alternative splicing pattern of certain transcripts [2,16]. Thus, splicing ratio changes, leading to differential abundance of splicing factor isoforms, could enhance the flexibility of the spliceosome composition and the splicing process itself. Hence, we asked whether this is the case for the genes shown to have an alternatively spliced NAGNAG. We experimentally tested several organs, developmental stages and environmental influences. Interestingly, significant organ-specific differences of splicing ratios were detected in four cases. Most notably, inflorescence showed reduced splicing of the minor acceptor in nearly all experimental candidates. A very similar effect was seen in early developmental stages (3d compared to later stages). A common reason may be that stem cells, enriched in both these samples, disfavor minor acceptor usage. This should be further tested in future experiments. Finally, the most pronounced effect on the splicing ratio was seen after cold shock, consistent with previous observations [21,47].

For the analyzed gene family, it seemed reasonable to ask for the impact of NAGNAG splicing on the RRM domain. We found that none of the eight NAGNAG acceptors in SR proteins do affect the RRM. In contrast, 12 of the 23 SR-related proteins have a NAGNAG acceptor located in the RRM domain. Previously, functional differences due to a NAGNAG acceptor in the RRM were observed for the U11–35K protein [42] and, more generally, NAGNAG alternative splicing in RRM-containing proteins was suggested to have an impact on the tertiary structures [41]. Also the usage of the E-acceptor site results in a protein with one additional serine in *SR33/SCL33 *and *RS41*. Serine residues in SR proteins are the targets of phosphorylation, and numerous studies have shown that the phosphorylation status of SR proteins is critical for their splicing activity as well as subcellular localization [2,14,16,27].

Most notably, the pattern of differential splicing ratios is similar for all analyzed genes. Thus, the differential effects on NAGNAG alternative splicing seem to be organ- and condition-specific rather than gene-specific. This favors the hypothesis that differential splicing of NAGNAG acceptors is mostly independent of sequence-specific splicing regulators, and is rather mediated by (subtle) organ- and condition-specific differences of the spliceosomal core composition. Intuitively, such lack of tight regulation seems to argue against a functional relevance of splice variants, as was suggested earlier [44]. However, several tandem splice sites with clear functional implications exhibit constant splicing ratios. Vice versa, it was shown that alternative splicing events producing variable splicing ratios do not always imply a function [48]. Surely, the functional relevance of the alternative splice events analyzed in this study remains to be evaluated.

## Conclusion

We demonstrated, that NAGNAG acceptors frequently occur in the Arabidopsis genome and are particularly prevalent in SR and SR-related protein-coding genes. Insufficient EST coverage can be compensated using the sequence-based method to predict alternative splicing of NAGNAG acceptors. The observed differential effects on NAGNAG alternative splicing appear to be organ- and condition-specific rather than gene-specific. In particular, inflorescence and early seedling stages consistently show reduced levels of the minor transcript isoforms.

## Methods

### Screening for NAGNAG acceptor tandems

The annotated genome sequence of *Arabidopsis thaliana *was obtained from GenBank, to serve as a data basis for the locations of intron-exon boundaries and their sequence. Boundaries with the sequence NAGNAG| or NAG|NAG (where "|" indicates the annotated boundary) were sampled. Redundancies due to annotation of multiple transcript isoforms were filtered. Potential splice variants derived from the genomic NAGNAG patterns were detected and quantified by a WU-BLASTN search of 60-nt sequence windows around the resulting exon-exon junctions against all Arabidopsis ESTs from TIGR and RIKEN databases [49,50], using parameters W = 13 N = -8 nogap S = 180 hspmax = 1. BLAST matches were considered valid if perfect sequence identity was found in a 12-nt window around the exon-exon junctions [51].

### Prediction of splicing ratios

All NAGNAG-containing introns with supporting EST data for E- and I-acceptor were divided into subsets of 64 motif classes, according to the heptameric motif NAGNAGN. Maximum-likelihood estimators for E-to-I transcript ratios were calculated by combining the EST counts per class. In order to prevent a bias caused by cases with an extremely high EST coverage, counts were limited to a maximum of 10 per isoform per NAGNAG site, and eventually downscaled.

### Identification of SR/SR-related protein genes

The complete set of non-redundant Arabidopsis proteins was screened for existing RNA-recognition motifs, and its derivatives, using Pfam HMM definitions (PF00076, PF04059, PF08777) and hmmsearch (HMMer package, [52]) applying recommended cutoff parameters. Additionally, the relative content of RS or SR dipeptides of each gene product was determined. A significance threshold >0.016 for R/S-richness was applied, corresponding to the transition point of a two-exponential case distribution. A subset of 84 proteins had both significant RRM profile hits and R/S-rich sequence. Of these 84, 19 are identified as SR proteins *sensu strictu *[18,19].

### Plant material and stress treatments

*A. thaliana ecotype Columbia *seedlings were grown on Murashige and Skoog (MS) medium at 22°C with 16 h/8 h light/dark cycle and harvested after 3d, 5d, 10d and 15d. Callus tissue was generated from roots of one-week-old seedlings by transferring them onto a callus induction medium (1× Gamborg's B5 medium, 2% glucose, 0.5 g/l MES (pH 5.7), 0.8% agar, 0.5 mg/l 2,4-D [2,4-Dichlorphenoxyacetate] and 0.005 mg/l kinetin). Callus tissue was collected and frozen in liquid nitrogen for RNA extraction. Heat and cold stress treatments were done with 15d-old seedlings. Seedlings were grown for 15-days and exposed to heat (38°C) for two and six hours or cold (4°C) for 12 and 24 hours, the untreated control seedlings were kept at 22°C for the corresponding time period.

### RT-PCR and splice-variant analysis by quantitative capillary electrophoresis

RNA from plant tissues, seedlings or callus was isolated using RNeasy Plant Mini Kit (Qiagen) and quantified spectrophotometrically at 260 nm. RNA was treated with DNAseI and used to synthesize first strand cDNA with oligo (dT) primer using SuperScriptII (Invitrogen). For validation of splice variants, three independent RT-PCRs for each candidate were performed with cDNA from different organs, developmental seedling stages and stress treatments to yield amplicons covering the respective exon-exon junction. Reactions were set up with BioMix Red (Bioline, Randolph, USA) and 10 pmol primer in 50 μl total volume, according to the manufacturer's instructions. Each forward primer was labelled with 6-carboxyfluorescein (FAM) for subsequent analysis on a capillary sequencer. The thermocycle protocol was 1 min 30 sec initial denaturation at 94°C, followed by 35 cycles of 50 sec denaturation at 94°C, 45 sec annealing at 55–59°C, 1 min extension at 72°C, and a final 1 h extension step at 72°C. The following gene-specific primers were used: *RS41 *reverse GCTGGCGGCGAACGAGA, *RS41 *forward GAGAAGGGAAAGCAGGAGTC, *SR33 *forward GCTGCTGATGCAAAACATC, *SR33 *reverse CTCCCATCATATCGCTCTTC, *SRZ22a *forward CGTGGTGGTTCTGATTTGAAG, *SRZ22a *reverse GATCTAGCACGAGGGCTGTAA, *SR45i9 *forward GTCGCTCTCGTTCAAGTTCC, *SR45i9 *reverse TTTACGAGGTGGAGGTGGTG, *SR45i7 *forward AGGCCGTTCTCCATCTTCTC, *SR45i7 *reverse CCTTCTGGGACTTGGTGAAC, *At2g24350 *forward CTGCGCTCTGTCATTGTTTC, *At2g24350 *reverse ACATGAGGCTCCGTTTCTTG, *At1g53650 *forward AGTTCTTCGCTTTGCGTTTG, *At1g53650 *reverse GCAGGCAGACTGAAAGAAGG, *At5g09880 *forward GGAAGAGAAGGAACCCGAAG, *At5g09880 *reverse CCATTGGAACTGACATCACG, *At5g59950 *forward TGGATGGAAAACCCATGAAG, *At5g59950 *reverse ACCACCTCGTTGTTGACCTC, *At1g76940 *forward ACATCATCCTCCTGGTGGTC, *At1g76940 *reverse CCACCTTCTCCTGATTGCAC, *At2g43370 *forward GGAGCTTCACGAGGATATGG, *At2g43370 *reverse CTCAGGCGGAAGCTGAATAC, *At4g35785 *forward ATCTCCTTCACCCCGAAAAG, *At4g35785 *reverse CAAGACGCAACCTTTCCTTC, *At1g60900 *forward GCGCCTCCTGATATGTTAGC, *At1g60900 *reverse AGGCCACCAACATAGACTCG, *At3g54230 *forward GGGTCCTTTGCATCATGTTC, *At3g54230 *reverse ACATCCGCTGAAGGAGAATC. The PCR products were appropriately diluted (1/20 to 1/50) and 1 ul was supplemented with 10 ul formamide (Roth, Karlsruhe) and 0.5 ul of GeneScan 500 LIZ (Applied Biosystems). The mixture was than separated on an ABI 3730 capillary sequencer and analyzed with the GeneMapper 4.0 software. The E-transcript proportion (%) was calculated as follows: peak area for the E-isoform/(E-isoform+I-isoform) × 100.

### Splice-variant analysis by clone-counting

For validation of splice variants (*RS41, SR33, SRZ22a, SR45*), RT-PCR was performed with cDNA from root, leaf, stem and inflorescence to yield amplicons covering the respective exon-exon junction. The following gene-specific primers were used: *RS41 *forward 5'-AAG AGG AGG GAA AGC AGGAG-3' and reverse 5'-GCG ATT TCG AAT GGA GTC AT-3'; *SRZ22a *forward 5'-GCA AGA ATG GAT GGA GGG TA-3' and reverse 5'-CCA CGA GGA GAA GGA CTA CG-3'; *SR33/SCL33 *forward 5'-AGG GTT TGG GTT CGT TCA AT-3' and reverse 5'-CTC CGT GAC CGA GAT CTA CC-3'; *SR45 *forward 5'-CAC CTC CAA GGA GAC TAC GC-3' and reverse 5'-CAG TGG CCT CTT AGG ACT GC-3'. PCR products were gel purified using the QIAquick Gel Extraction Kit and the isolated fragments were cloned into pCR2.1-TOPO (Invitrogen) according to the supplier's recommendations. 25 clones per gene and plant sample were selected and Sanger-sequenced using M13 standard reverse primer (20-mer). Sequence analysis was performed using SPIDEY [53].

## Authors' contributions

SS performed the capillary electrophoresis experiments, analyzed and interpreted data and wrote the initial manuscript. KS conceived and designed experiments, performed the statistical analyses, interpreted data and contributed to the manuscript. MH performed the genome-wide screening. GSA and SGP performed experiments, analyzed and interpreted data. ASNR, RB and MP as principal investigators conceived the experiments. All authors contributed to the final manuscript.

## Supplementary Material

Additional file 1 **All NAGNAG acceptor cases identified within the Arabidopsis genome**. The absolute numbers of ESTs supporting the E- or the I-acceptor ("ESTs_E" and "ESTs_I", respectively) and the sequence-based prediction of E-transcript frequency ("expected_E") are given. Click here for file

Additional file 2 **Counts of Arabidopsis NAGNAG cases depending on local EST coverage**. The number of cases at least reaching a given coverage is presented. Click here for file

Additional file 3 **Heptamer motif classification**. Heptameric motif classes are presented with corresponding maximum-likelihood estimators for E-to-I-transcript ratios, used for sequence-based prediction. Click here for file

Additional file 4 **Comprehensive list of experimental values from all analyzed SR and SR-related protein-coding genes**. Column 'avg' lists the averaged E-transcript proportions derived from three independent experiments per probe with corresponding standard deviation in column 'sd'. The values derived from the 3d, 5d, 10d, 15d old seedlings, callus and organs were averaged (column 'avg organs-seedlings') as well as the corresponding standard deviations ('avg error') to gain appropriate values for a comparison with the sequence-based predictions (see column 'predicted'). Grey shaded values mark the cases where NAGNAG alternative splicing was validated [(avg error × 2) < (avg organs-seedlings)]. Click here for file
